# Regulation of Immune Reactivity by Intercellular Transfer

**DOI:** 10.3389/fimmu.2014.00112

**Published:** 2014-03-28

**Authors:** Maxime Dhainaut, Muriel Moser

**Affiliations:** ^1^Laboratory of Immunobiology, Université Libre de Bruxelles, Gosselies, Belgium

**Keywords:** T/APC interaction, trogocytosis, T cell activation, costimulation, immune regulation

## Abstract

It was recently proposed that T lymphocytes, which closely interact with APCs, can extract surface molecules from the presenting cells when they dissociate. These observations question the classical view of discrete interactions between phenotypically defined cell populations. In this review, we summarize some reports suggesting that membrane exchange at the immune synapse can be a vector for intercellular communication and envisage some consequences on the biology of T cells.

## Introduction

The first evidence that activation of T and B lymphocytes required a co-operation between distinct cell types was provided in the late 60s. In 1967, Mosier showed that both adherent and non-adherent cells were necessary for the induction of antibody formation to sheep red blood cells *in vitro* (1). A few years later, it was shown that recognition of soluble protein antigens by guinea pig T lymphocytes required the presentation of antigen on histocompatible macrophages (2). Since then, numerous observations have highlighted the multiple interactions, which occur at various steps of the immune response: in particular, antigen-presenting cells are likely to provide three signals to T lymphocytes, which in turn trigger antibody production by B cells. These cell populations are located at discrete sites in lymphoid organs and migrate to particular sites to interact with each other. The immune response appears therefore as an exchange of signals between cells displaying well-defined phenotypes, and the specificity of the interaction is ensured by receptor/ligand interaction or binding of soluble cytokines on their receptor.

However, recent observations may challenge this scenario. Indeed, there is increasing evidence that intercellular transfer of membrane fragments and molecules occurs frequently during cell–cell close contact, thereby modifying the phenotype and probably the function of immune cells. This process has been named “trogocytosis,” from the ancient Greek *trogo*, meaning “gnaw.” In this report, we will review recent observations illustrating membrane exchange between immune cells, focusing on T cells and antigen-presenting cells, and envisage the possible physiological consequences of this phenomenon.

## TCR MHC/Peptide Complex

A number of old reports have documented the existence of T cells bearing IA antigens on their membrane, at a time when MHC restriction was unclear and the T cell receptor unidentified. In particular, Nepom et al. showed that I-A^+^ T cell blasts appeared in antigen-stimulated proliferative culture, and that this acquisition was strictly antigen-dependent and required positive adherent antigen-presenting cells (3). Subsequent reports confirmed that T cells may acquire peptide/MHC complexes at the T cell–APC interface. Huang et al. showed that these complexes on APCs formed clusters at the site of T cell contact within minutes, and were subsequently acquired and internalized in T cells (4). The intercellular transfer of membrane molecules was also observed *in vivo* in several models: rat T cells transferred in irradiated SCID mice acquired MHC molecules as well as adhesion and costimulatory molecules (5), and encephalitogenic T cells were shown to express abundant surface MHC class II molecules in rat and mouse models of EAE (6). In addition, in the course of studies aimed at understanding the affinity maturation of secondary T cell responses, Kedl et al. (7) provided evidence for a mechanism of stripping of antigen/MHC complexes by T cells. The interaction of antigen-specific T cells with the APCs *in vivo* induced the selective loss of the antigen–MHC ligand from the surface of DCs. Another report describes the transfer of specific GFP–MHC–peptide complexes from transfected fibroblasts to T cells. Among T cells interacting with transfected fibroblasts, about 10% spontaneously dissociated within about 10 min and acquired GFP-labeled complexes from the immunological synapse. The intercellular transfer was peptide-specific and -correlated with the activation state of the T cell, as assessed by CD69 expression (8). Acquisition of membrane molecules from APCs seems to be an inherent feature of activated CD4^+^ T cells, and continues during cell cycle progression (9). Of note, T helper cells and regulatory T cells have a comparable capacity of trogocytosis *in vivo*, as demonstrated by the similar acquisition of MHC II by CD4^+^CD25^−^ (helper T cells) and CD4^+^CD25^+^ (regulatory T cells) cells from HA-transgenic mice adoptively transferred into Balb/c mice followed by immunization with HA (9). Finally, a recent report demonstrates that MHC II was displayed on the surface of TCR transgenic CD8 T cells activated *in vitro* with the cognate peptide. Notably, in mice infected with LCMV Arm i.v., up to 25% of viral peptide-specific CD8^+^ T cells displayed MHC II on their surface. Among the three major populations of APCs, DCs transferred the most MHC-II onto CD8^+^ T cells (10).

## Costimulatory Signals

In addition to the appropriate antigenic signal, APCs may provide costimulatory signals, which are required for optimal activation of naive T cells. Several ligand/receptor pairs have been described, which potentialize the signal induced via the TCR. In particular, signaling downstream of the CD28 receptor on T cells positively regulates proliferation and survival of T cells, as well as their cytokine production (11). The first evidence that B7 ligands could be taken up by T cells was provided by Hwang et al. who showed that rat T cells acquired murine CD80 and CD86, both *in vitro* when co-cultured with murine DCs or *in vivo* when transferred into irradiated SCID mice (5). This acquisition was under the control of either CD28–B7 or TCR–peptide–MHC interaction: indeed, CD28^−/−^ T cells cultured with DC displayed a 10-fold reduced expression of MHC II and CD80, as compared to CD28^+^ T cells. Subsequent studies confirmed these observations in mice, using cocultures of DCs and CD86/CD80 double knock-out T cells (12). The acquisition was directly related to the strength of signals 1 and 2. Interestingly, the observations suggest a different outcome in naïve versus memory T cells: naïve T cells became capable of acting as APCs, whereas memory T cells underwent increased apoptosis.

## Physiological Consequences

### Positive regulation

Although the physiological consequences of the intercellular transfer are still questionable, several observations suggest an active role in the immune responses. (i) Kedl et al. concluded that T cells may compete with each other by lowering the amount of antigen–MHC complexes on the APCs, and showed that their ability to compete was affected by their affinity for the MHC/antigen complexes, thereby driving the affinity maturation of memory T cell responses (7). These data provided a mechanism for their previous observation that competition between T cells of the same peptide–MHC specificity occurred efficiently *in vivo* (13); (ii) The transferred antigen–MHC complexes appeared associated with molecules that imply continuous signaling, namely the src family kinase p56lck and tyrosine-phosphorylated proteins. The sustained signaling may be required for full activation of T cells even when contacts with DCs are of short duration (8); (iii) CD8^+^ T cells have been shown to acquire MHC class II molecules *in vitro* and *in vivo* in response to viral infection, a transfer which conferred to them the capacity to directly activate CD4^+^ T cells. The direct CD4/CD8 T cell interaction may contribute to help for CD8^+^ T cells and provide an alternative model to the DC licensing or the three cell cluster (10); (iv) the intercellular transfer of antigen–MHC complexes may expand the repertoire of cells that can function as APCs, and regulate an ongoing immune response. This hypothesis would be consistent with a recent report (14) showing that differentiation of CD8^+^ T cells required not only T cell–APC interactions but also T cell–T cell synapses. The authors showed that these T cell interactions promoted critical synaptic cytokine exchange, allowing CD8^+^ T cells to share IFN-γ for example, and interpret their data as a collective decision-making resulting to positive reinforcement. However, it is possible that, in addition, these synapses could mediate antigen-specific signaling through the captured peptide–MHC complexes.

### Negative regulation

Conversely, intercellular transfer may downregulate immune responses. There is some evidence that the presence of APC-derived peptide/MHC complexes on T cells may render them susceptible to fratricide lysis. Huang et al. have indeed shown that T cells cultured with APCs for 1 h were susceptible to lysis provided a high density of peptide/MHC complexes was transferred (4). Another report confirms that triggering of fratricide required extremely high levels of antigenic peptides (15), suggesting that this mechanism of exhaustion would occur in the presence of high antigen concentration, i.e., in certain viral infections.

A few studies revealed an interesting correlation between anergy induction and T cell-mediated APC activity (16–18). Adoptive transfer of MBP-pulsed transformed T cells (expressing high levels of MHC II, CD80, and CD86) resulted in reduced severity of EAE in naïve rats (17), whereas mouse CD4^+^ T cells, which have acquired MHCII/peptide complexes were susceptible to apoptosis and hyporesponsive to the antigen pulsed on mature dendritic cells (18). These observations suggest that T cells may present peptide–MHC complexes in a tolerogenic manner.

It is likely that the nature of the cell that has acquired antigen/MHC would determine the consequence of trogocytosis. In particular, double-negative Tregs have been shown to acquire alloantigen *in vivo*, allowing them to specifically kill syngeneic CD8^+^ T cells that can interact with the alloantigen (19). The outcome of trogocytosis by T helper versus regulatory T cells (following coculture with antigen-pulsed A20 cells) was different, with T helper cells able to drive activation of naive CD4^+^ T cells and Treg displaying an enhanced suppressive activity (9).

A few reports suggest a cross-regulation between a receptor and its ligand, which could involve intercellular transfer of either molecule. The analysis of ICOS-Tg mice revealed unexpectedly a phenotype resembling ICOS-deficient mice, i.e., reduced titers of IgG1 and IgE in serum and attenuation of germinal center formation. The defect of ICOS-Tg mice in antibody production was not due to an intrinsic defect of T or B lymphocytes but rather to a defect in the *in vivo* environment. It was further shown that APCs displayed reduced ICOSL expression (at the protein but not the mRNA level), suggesting a negative feedback regulation by ICOSL downregulation in response to ICOS expression (20). Similarly, Kuka et al. studied mice deficient in either CD27 or CD70 and found that CD27 and CD70 cell-surface expression was reciprocally regulated. When CD27 was blocked, CD70 transcripts increased more than 300-fold, indicating that the interaction of CD27 with CD70 inhibits CD70 transcription (21). Our own studies revealed a distinct mechanism of regulation, as we found that thymus-derived Tregs and activated T cells inhibited CD70 expression on DCs at the protein level, by a mechanism that involves transfer of intact CD27 from the T cell to the DCs (Dhainaut et al., submitted). Collectively, these observations highlight a reciprocal regulation of a unique ligand/receptor pair, which may provide rapid fine-tuning of ongoing T cell responses.

## Human Studies

A few reports suggest that a similar acquisition of membrane molecules may occur in humans. Human T cells cultured with DCs acquired CD80, and the level of “expression” was related to the level of CD80 expression on APCs and was enhanced upon TCR engagement (by anti-CD3 mAb or alloMHC recognition). The transfer of CD80 to T cells was mediated by its receptor, as blockade with soluble fusion proteins (sCD28, sCTLA-4, and sCD80) prevented its acquisition, and resulted in T cells able to provide costimulatory signals (22). Another report confirmed these observations and showed that T cells could acquire HLA-DR and B7 molecules from DCs during an alloresponse and then acted as APCs to resting autologous T cells (23). In addition to CD28/CTLA-4 and their ligands, other receptor ligand pairs can provide costimulatory signals to T cells. Baba et al. showed in humans that the intact OX40L molecule was transferred from APC to T cell, in various cell combinations, in a contact dependent manner. The transferred OX40L was functional and displayed as discrete punctate pattern on the T cell surface (24).

T cells can also be imprinted by tumor antigen. A high proportion (ranging from 10 to 70%) of melanoma specific T cell clones were shown to acquire tumor antigens *in vitro* and this transfer could be used to identify tumor antigen-specific T cells in patients. Thus, freshly isolated tumor-infiltrating lymphocytes expressed melanoma antigens and the tumor antigen imprinting correlated with antitumor T cell function. Indeed, tumor antigen-imprinted CTL exhibited superior killing activity, suggesting that the antigen acquisition may enhance their effector function (25, 26).

## Molecular Mechanism of Acquisition

Martinez-Martin et al. have examined the mechanism of TCR internalization at the immunological synapse and showed that it was coupled to the TCR-triggered acquisition of membrane fragments from the antigen-presenting cell (27). They further showed that two Ras family GTPases, TC21 and RhoG, which colocalize with the TCR at the immune synapse (28), mediated internalization of the TCR via a clathrin-independent endocytosis. The authors interpret the process as an incomplete phagocytosis of the whole APC by the T cell, which results in the removal of an APC fragment. Whether the TCR and the trogocytosed APC membrane fragments that include MHC complexes are recycled or degraded is an important question, as it would have opposite impact on the immune response. TC21- and RhoG-deficient T cells showed increased responsiveness to TCR stimulation, suggesting that TCR downregulation (and possibly acquisition of APC fragments) could be involved in the termination of the response.

As peptide/MHC complexes, costimulatory, and adhesion molecules appear to be co-transferred to T cells (5), it is likely that the mechanism described by Martinez-Martin et al. could be a common mechanism for membrane exchange at the immune synapse. The strength of the interaction, which results from several ligand/receptor interaction, seems to determine the amount of membrane fragments transferred. Accordingly, CD28-deficient T cells exhibited less stable interactions with APCs in cocultures and absorbed less MHC molecules than CD28 competent T cells (5), and the acquisition of CD80 was directly correlated to the strength of signal 1, i.e., the concentration of antigenic peptide (12). In addition to trogocytosis (which allows rapid transfer of intact surface molecules by phagocytosis probably at the immune synapse), other mechanisms exist which involve transfer of various types of vesicles with a slower kinetics [for review, see (29, 30)]. The respective contribution of both mechanisms remains to be determined but could be dependent on the nature of the cells, their state of activation, and the microenvironment.

## Conclusion

**Figure 1:**
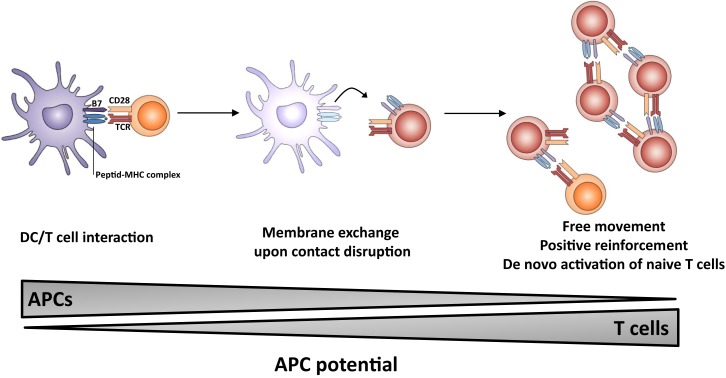
**Proposed model for the role of membrane exchange in T cell activation**. The first step involves close interaction between APC and T cell (left panel) and acquisition of MHC and costimulatory molecules by T cells upon dissociation (middle panel). The second step involves presentation of antigen and costimulatory molecules by T cells, leading to sustained activation (and possibly naïve T cell priming) in the absence of conventional APCs.

The outcome of the process of membrane exchange remains elusive but could lead to an enhancement of the resulting immune response (Figure 1). In particular, T cells which have acquired molecules from APCs may gain some capacity of antigen presentation, thereby (i) multiplying the number of cells presenting the antigen, (ii) prolonging the presentation step in the absence of DC/T interaction possibly outside lymphoid organs, i.e., in peripheral tissue; (iii) allowing T cells to move freely and interact with effector lymphocytes (B cells and CTL). It is of note that T lymphocytes do probably display a higher lifespan than dendritic cells. Thus, the membrane exchange would result in sustained autonomous activation without requirement for prolonged T-cell interaction between DC, CD4 T helper, and effector cell. Collectively, these observations highlight the multiple roles of the immunological synapse, which appears to trigger membrane-bound receptor–ligand interactions, cytokine release as well as membrane exchange.

## Conflict of Interest Statement

The authors declare that the research was conducted in the absence of any commercial or financial relationships that could be construed as a potential conflict of interest.
